# Case of left atrial myxoid sarcoma with lung metastases presented as a cardiogenic shock: a case report and literature review

**DOI:** 10.1097/MS9.0000000000000898

**Published:** 2023-06-05

**Authors:** Omar R.S. Khalil, Belal M.M. Omar, Lamees Khalil, Doaa Tarabieh, Layth Al-Karaja, Hasan Alkhatib, Diya Asad

**Affiliations:** a Faculty of Medicine, Al-Quds University; b Department of Adult Cardiac Surgery, Al-Makassed Charitable Hospital, Jerusalem, Palestine

**Keywords:** cardiac sarcoma, case report, metastases, thoracic surgery

## Abstract

Cardiac tumors are a rare condition that typically presents with nonspecific symptoms. Among the histologic patterns, myxoid sarcomas are rarely identified and may have a less favorable prognosis. Reporting a case of this type of cardiac tumor can increase awareness about this condition and aid in early diagnosis, potentially leading to better outcomes.

**Case presentation:**

We are presenting a case of a 41-year-old female with left atrial myxoid sarcoma, which was presented with a cardiogenic shock picture. She underwent surgical excision of the mass and was discharged in good condition. After discharge, she deteriorated and was found to have lung metastases.

**Clinical discussion:**

Primary cardiac sarcomas, due to their rarity and poor prognosis, are often diagnosed at an advanced stage of the disease and lack sufficient data to establish a standard course of treatment. The cornerstone of therapy is surgical resection. However, novel therapeutic approaches must be developed.

**Conclusions:**

Primary cardiac tumors should be suspected in adult patients with progressive dyspnea, and a biopsy should also be done to determine the histopathological pattern of the mass and estimate the overall prognosis and outcomes.

## Introduction

Highlights

- Myxoid sarcoma is a rare type of cardiac tumor that may be misdiagnosed as a myxoma.
- Myxoid sarcoma has high metastatic capacity, most likely to the lungs.
- Echocardiogram is the gold diagnostic tool, computed tomography or positron emission tomography scans for extension and metastases.
- Surgical resection is the standard of care, and the prognosis depends on metastases.

The majority of primary cardiac tumors are benign and infrequent. Myxomas, which have an estimated postmortem incidence of 0.375–11.25 per 100 000 cases, are the most prevalent benign cardiac tumors, while sarcomas, which have an estimated autopsy incidence of 0.275–7.125 per 100 000 cases, are the most prevalent malignant cardiac tumors^1^. Clinically, it can be challenging to differentiate between benign and malignant tumors because symptoms are frequently not related to the type of tumor but rather to its location in the heart^2^. Primary cardiac sarcomas can develop at any age, with a mean age of 41 years and a higher incidence during the third and fifth decades^3^.

Surgery continues to be the mainstay of care for these patients’ local control and survival. Nonetheless, considering the use of adjuvant therapies on an individual basis is important, especially when the condition is unresectable. With a median survival of 6–12 months, the prognosis is still poor. Full surgical resection has improved survival when paired with radiation therapy and preoperative chemotherapy^4,5^. Herein we are presenting a case of a 41-year-old female with left atrial myxoid sarcoma, which was presented with a cardiogenic shock picture. She underwent surgical excision of the mass and was discharged in good condition. After discharge, she deteriorated and was found to have lung metastases. This case has been reported in line with the 2020 SCARE (Surgical CAse REport) guidelines^6^.

## Case presentation

### Patient information and clinical findings

A 41-year-old female of Middle Eastern ethnicity was presented to the emergency department in September 2018 by her family via ambulance. The patient was unemployed and had a past medical history of left leg fibro-osteoblastic sarcoma since 2015, which was treated with chemotherapy and radiotherapy. She had no other significant medical history and was not on regular medications other than painkillers. She also had an above-the-knee amputation of the left leg. There is no relevant family history reported. The patient is a nonsmoker and uses a wheelchair. She was complaining of a 3 days duration of shortness of breath associated with chest tightness, dry cough, progressive orthopnea, and paroxysmal nocturnal dyspnea. She has no associated chest pain, palpitations, or lower limb edema. On examination, she was conscious, oriented, and alert but in respiratory distress. She was desaturated with SpO_2_ =91, her heart rate (HR) =130, her respiratory rate (RR) =30, and her blood pressure (BP) =90/50 with good air entry bilaterally, on auscultation. She has bronchial breathing on the right lower zone and bilateral basal crepitation, her jugular venous pressure was not elevated, and she has no lymphadenopathy or breast masses.

### Diagnostic assessment

After primary assessment with a thorough history and physical examination, an ECG was done and showed sinus tachycardia, X-ray showed bilateral apical infiltrate with air bronchogram, lower limb Doppler showed no deep venous thrombosis, her D-dimer was negative, and the labs including complete blood count showed WBC of 19 000 with a shift to the left, creatinine (Cr) =1.7 and troponin =30. She was transferred to the intensive care unit to keep under observation as she was desaturated and her blood pressure was low, and she was given levofloxacin, ceftriaxone, and sulfamethoxazole–trimethoprim because pneumonia was suspected, and septic work-up was done with pending results.

**Figure 1:**
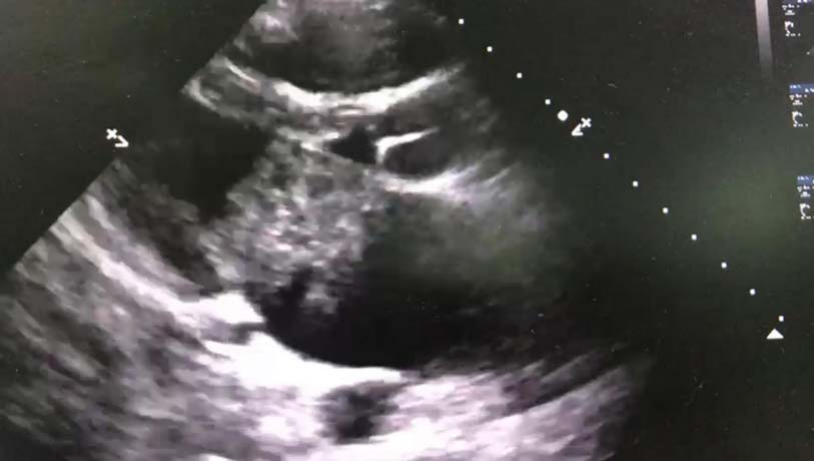
The mass as appeared on cardiac echo.

**Figure 2:**
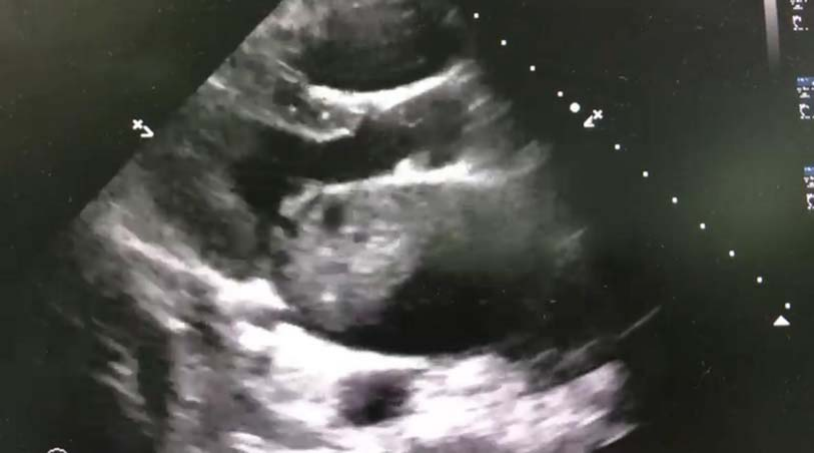
The mass as appeared on cardiac echo.

On the second day of admission, her condition deteriorated; a repeat chest X-ray showed a bilateral apical infiltrate suggestive of acute respiratory distress syndrome (ARDS), and she was intubated. Her mechanical ventilation settings, tidal volume (TV) =380, positive end-expiratory pressure (PEEP) =10, fractional inspired O_2_ (FiO_2_) =100%, and RR =16. Her vitals include BP =90/60, oxygen saturation =88%, and HR =130 despite her being on a maximum dose of norepinephrine of 30 μg. RR was increased to 20, PEEP increased to 14, and given 1000 cm^3^ running intravenous (i.v.) fluid. Her saturation improved to (99%) and she planned to decrease FiO_2_ and PEEP according to saturation. But her BP still did not improve; an urgent echocardiogram was done and showed a left atrial mass (Figs 1, 2).

### Therapeutic intervention

**Figure 3:**
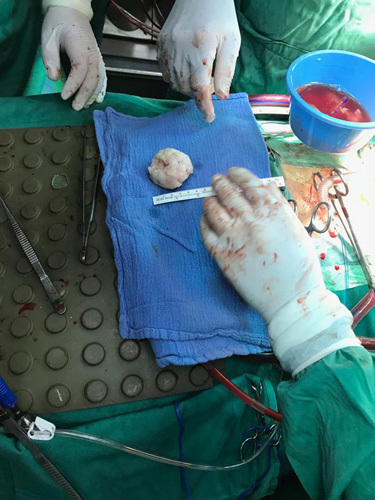
The excised tumor after surgery.

After the echocardiogram findings, the Cardiothoracic team consulted urgently and advised urgent surgery. The decision to proceed with the surgery was made after consulting the anesthesiologist and the cardiologist. The surgery was performed by a highly skilled and experienced cardiothoracic surgeon, who was assisted by a full staff of nurses, anesthesiologists, and other medical professionals. During the surgery, the chest was opened by a mid-sternotomy incision. After heparinization, aortic and bicaval cannulation was performed. Cooled blood antegrade cardioplegia was given after cross-clamping the ascending aorta. The left atrium was opened, and a huge tumor measuring 6×6 cm was resected en bloc (Fig. 3). The mitral valve was tested and showed no significant regurgitation, then after closing the left atrium and de-clamping the aorta, the heartbeat was sinus. De-airing was done with transesophageal echocardiography (TEE) guidance, and the patient was disconnected from the bypass after warming with high inotropic support. TEE showed mild mitral regurgitation with good left ventricle function and no residual masses. A biopsy was taken and showed myxoid sarcoma.

### Follow-up and outcomes

On the day of surgery, the patient was in critical condition on noradrenaline, adrenaline, milrinone, and dopamine, and then we started weaning until we reached 10 μg of norepinephrine, and her situation improved and she became hemodynamically stable with a mean arterial pressure of 80. She was on mechanical ventilation: TV =500, RR =22, and FiO_2_ =50%. A chest X-ray post-operation showed bilateral patchy opacities. She was kept on meropenem and sulfamethoxazole–trimethoprim. She was extubated in the morning of the second day post-operation; her vital signs were good without O_2_ support, and she had a good urine output. After 11 days of the surgery, the patient was in good general condition with a healthy dry sternal wound, so she was discharged with full instructions and to continue her management with the oncology center for possible chemoradiotherapy.

After we discharged her, she did a chest computed tomography, which showed metastatic lung nodules. So chemotherapy was planned. Ten days after her discharge and before her chemotherapy session, her doctor referred the patient to us because of a turbid, moderate discharge from the sternal wound, which was treated successfully. Three months after her second discharge, she developed a general weakness of 2 days duration associated with loss of appetite, diarrhea without blood or mucus, and cough. She had no history of fever or vomiting; therefore, she was brought to the emergency room. On examination, she was pale, not responsive, and had pale extremities; she also had shallow breathing with oxygen saturation of 70% and tachycardia with HR of 137. She was managed as a case of hypovolemic shock initially, two i.v. lines were applied and given 1000 cm^3^ of normal saline; afterward, she had a BP of 80/60. Because of her low oxygen saturation, she was given oxygen via a facemask, and then her oxygen saturation went up to the 80s. Unfortunately, despite the resuscitative efforts, the patient died.

## Discussion

Sarcomas are diverse, clinically challenging tumors of the soft tissue and bone. Sarcomas are the second most prevalent type of solid tumor in children and adolescents and make up a significant subset of secondary malignancies, although makes up only 1% of all human malignancies. To date, more than 100 histological subtypes have been identified, and molecular profiling is revealing many more. Several sarcoma subtypes fall into the most difficult-to-treat categories due to their generally aggressive biological behavior, relative rarity, and prevalence at almost every anatomical site^7^. Autopsies only reveal primary cardiac tumors in 0.001–0.3% of cases, making them extremely rare^8,9^. Around 75% of primary heart tumors are benign, and 25% are malignant, and sarcomas make up around 75% of the malignant tumors^8^. Whereas the reported incidence of metastatic cancers, which ranges in published data from 1.5 to 20%, is 20–40 times higher than primary neoplasms^10^. The most prevalent histological subtypes include lymphomas, mesothelioma, fibromyxosarcoma, angiosarcomas, and rhabdomyosarcomas^10^. This variety in histological subtypes and morphology is attributed to their mesenchymal origin^8^.

Malignant cardiac tumors are often asymptomatic until metastasis occurs and are typically detected incidentally on imaging studies^10,11^. However, when left atrial masses, whether benign or malignant, cause symptoms, dyspnea due to mitral valve obstruction is the most common presentation^12^. In our case, the patient presented with cardiogenic shock, which is an unusual presentation of a cardiac mass and a life-threatening condition requiring rapid and aggressive management. This presentation diverted our attention away from the possibility of a cardiac mass and delayed the proper diagnosis until further investigations were conducted. The literature suggests that the presenting symptoms of cardiac tumors may mimic those of other heart conditions and depend largely on the location of the tumor and the extent of myocardial invasion rather than histology^11^.

The initial clinical and radiologic characteristics of cardiac sarcomas could be confused with other benign cardiac masses, particularly cardiac myxomas, making the diagnosis particularly challenging^9,12^. Due to the similarities in presenting symptoms, it can be difficult to distinguish between benign and malignant heart tumors; nevertheless, differences mainly appear in histology and clinical course^9^. The presence of several masses, non-septal attachment of the mass, extensive attachment of the mass to the left atrial wall, extension into the pulmonary vein, and semisolid consistency are all clues that favor the diagnosis of sarcoma over myxoma^12^. The first-line imaging modality that provides critical data about mass location, shape, attachment, size, mobility, and hemodynamic impact is typically transthoracic or transesophageal echocardiogram (TTE and TEE)^3,10,12^.

A complete surgical resection, which is only possible in less than 50% of patients, is the ideal treatment strategy. The local extension may still make resection incomplete or even impossible. However, neoadjuvant chemotherapy may reduce the tumor burden, making large tumors more resectable^9^. Several experimental procedures are being applied due to the poor results of traditional therapeutic options. The most important of these is cardiac transplantation or autotransplantation, which can only be applied in cases of local invasion without distant metastasis, and their results are not yet fully understood^11^. The prognosis is quite poor, with a mean survival of 3 months to 1 year, due to the slow diagnostic process, the difficulties of treatment, and the high risk of metastasis^9,12^. Most sarcomas develop rapidly and kill their victims by widely invading the myocardium, obstructing blood flow through the heart, or spreading to distant organs^11^. Distant metastasis was reported in approximately half of the patients, with about 50% having lung metastasis, which made management much more challenging and decreased survival significantly^9^. Our patient was found to have lung metastasis after the resection of the primary tumor, so chemotherapy was started. However, her condition deteriorated rapidly, and she died after the second chemotherapy session.

## Conclusions

In conclusion, primary cardiac sarcoma is a rare but life-threatening condition that can present with nonspecific symptoms and be easily mistaken for other cardiac masses. A thorough diagnostic work-up, including a multiparametric imaging approach, is essential to detect and characterize these tumors accurately. Surgical resection remains the mainstay of treatment, but it may not always be possible due to the extent of local invasion or the presence of metastasis. Therefore, neoadjuvant chemotherapy can be used to downsize the tumor and increase the possibility of resection. While radiation therapy is still controversial, autotransplantation may be a promising alternative approach for selected cases. Conventional heart transplantation is reserved for those patients who have been evaluated thoroughly and have no evidence of metastasis. Early diagnosis and aggressive management are crucial for improving the prognosis of primary cardiac sarcoma.

## Ethical approval

The study is exempt from ethical approval in our institution.

## Consent

Written informed consent was obtained from the patient’s family for the publication of this case report and accompanying images. A copy of the written consent is available for review by the Editor-in-Chief of this journal on request.

## Source of funding

This research received no external funding.

## Author contribution

O.R.S.K., L.A.-K., and D.A.: study concept or design; O.R.S.K., B.M.M.O., L.K., and D.T.: writing the manuscript; O.R.S.K., B.M.M.O., and D.A.: review and editing the manuscript; D.A.: data collector; H.A.: supervision.

## Conflicts of interest disclosure

The authors have no conflicts of interest to disclose.

## Research registration unique identifying number (UIN)

Not applied.

## Guarantor

Omar R.S. Khalil.

## Data availability statement

Data supporting the study results can be provided, followed by a request sent to the corresponding author’s e-mail.

## Provenance and peer review

Not commissioned, externally peer-reviewed.
